# Serotype Distribution and Invasive Potential of Group B Streptococcus Isolates Causing Disease in Infants and Colonizing Maternal-Newborn Dyads

**DOI:** 10.1371/journal.pone.0017861

**Published:** 2011-03-21

**Authors:** Mashudu Madzivhandila, Peter V. Adrian, Clare L. Cutland, Locadiah Kuwanda, Stephanie J. Schrag, Shabir A. Madhi

**Affiliations:** 1 Vaccine Preventable Diseases and Respiratory and Meningeal Pathogens Research Unit, Department of Science and Technology/National Research Foundation, University of Witwatersrand, Johannesburg, South Africa; 2 Centers for Disease Control and Prevention, Atlanta, Georgia, United States of America; Columbia University, United States of America

## Abstract

**Background:**

Serotype-specific polysaccharide based group B streptococcus (GBS) vaccines are being developed. An understanding of the serotype epidemiology associated with maternal colonization and invasive disease in infants is necessary to determine the potential coverage of serotype-specific GBS vaccines.

**Methods:**

Colonizing GBS isolates were identified by vaginal swabbing of mothers during active labor and from skin of their newborns post-delivery. Invasive GBS isolates from infants were identified through laboratory-based surveillance. GBS serotyping was done by latex agglutination. Serologically non-typeable isolates were typed by a serotype-specific PCR method. The invasive potential of GBS serotypes associated with sepsis within seven days of birth was evaluated in association to maternal colonizing serotypes.

**Results:**

GBS was identified in 289 (52.4%) newborns born to 551 women with GBS-vaginal colonization and from 113 (5.6%) newborns born to 2,010 mothers in whom GBS was not cultured from vaginal swabs. The serotype distribution among vaginal-colonizing isolates was as follows: III (37.3%), Ia (30.1%), and II (11.3%), V (10.2%), Ib (6.7%) and IV (3.7%). There were no significant differences in serotype distribution between vaginal and newborn colonizing isolates (P = 0.77). Serotype distribution of invasive GBS isolates were significantly different to that of colonizing isolates (P<0.0001). Serotype III was the most common invasive serotype in newborns less than 7 days (57.7%) and in infants 7 to 90 days of age (84.3%; P<0.001). Relative to serotype III, other serotypes showed reduced invasive potential: Ia (0.49; 95%CI 0.31–0.77), II (0.30; 95%CI 0.13–0.67) and V (0.38; 95%CI 0.17–0.83).

**Conclusion:**

In South Africa, an anti-GBS vaccine including serotypes Ia, Ib and III has the potential of preventing 74.1%, 85.4% and 98.2% of GBS associated with maternal vaginal-colonization, invasive disease in neonates less than 7 days and invasive disease in infants between 7–90 days of age, respectively.

## Introduction

Group B streptococcus (GBS) has been identified as a major cause of neonatal infection since the 1970s [1], [2]. GBS acquisition by newborns from maternal recto-vaginal colonization is an established risk factor for GBS sepsis within the first 7 days of life [3]. Vertical acquisition of GBS, involving colonization of the skin or mucous membranes, occurs in 15% to 50% of newborns born to GBS colonized mothers. An estimated 1–2% of newborns colonized by GBS develop invasive disease [4], [5].

Maternal vaccination against GBS, aimed at possibly reducing maternal colonization and enhancing transplacental transfer of anti-GBS antibody to the foetus, is being explored to prevent GBS associated sepsis during early infancy [6], [7]. The success of maternal immunization in preventing young infant morbidity and mortality is best characterized by the success of tetanus immunization program and inactivated influenza vaccine studies during pregnancy [8], [9].

GBS serotype specific polysaccharide-protein conjugate vaccine candidates, including serotypes Ia, Ib and III, have been evaluated for safety and immunogenicity [6], [10]. These vaccines are expected to provide serotype specific protection, hence, the need to undertake serotype characterization of colonizing and invasive GBS isolates, and estimating the invasive potential of individual serotype. GBS serotyping is based on the identification of specific capsular polysaccharide (CPS) epitopes (Ia, Ib, II to IX) [11], [12]. There are limited data on GBS serotype epidemiology associated with recto-genital maternal colonization or invasive disease in infants from industrializing countries.

We aimed to: i. study the serotype distribution of GBS isolates associated with colonization in mother-newborn dyads; ii. characterize the capsular serotype distribution of invasive isolates from infants; and iii. compare the relative invasive potential of GBS serotypes associated with early-onset disease.

## Materials and Methods

### Ethics Statement

Analysis of the GBS isolates for this study was approved by the Human Research Ethics Committee on Human Subjects (HREC) at the University of the Witwatersrand. Signed informed consent for collection of the isolates was obtained from study participants of the PoPS study, which was also approved by the HREC. The PoPS study was registered in ClinicalTrials.gov (Trial number: NCT00136370).

### Study Population

The study was conducted at a secondary-tertiary care hospital (Chris Hani-Baragwanath Hospital; CHBH) which provides health care to an urban population of 1.5 million indigenous Africans living in Soweto, South Africa. There are approximately 28 000 births annually in Soweto, 75% are born at CHBH and the remainder at one of seven surrounding primary health care clinics. The majority of Sowetan children requiring hospitalization are admitted at CHBH.

GBS isolates from mother-newborn dyads were identified from participants involved in the Prevention of Perinatal Sepsis (PoPS) trial undertaken at CHBH between April 2004 to October 2007 as reported [13]. Briefly, mothers were randomized during active labor for intra-vaginal chlorhexidine washes (0.5%) or external genitalia wipes with water. Vaginal swabs were undertaken prior to initiation of any study procedure and swabs were obtained from the newborn's ears, nose and umbilicus shortly after birth. The swabs were transported in Amies medium and processed using standard microbiological assays as described [13]. The PoPS study indicated that intra-vaginal chlorhexidine wipes were not associated with any reduction in vertical acquisition of GBS in the newborns. [13] Serotyping in this study was, nevertheless, limited to mother-newborn dyads who had been randomized to the placebo arm of the PoPS trial.

In addition, prospective surveillance of early infant sepsis at CHBH was undertaken from January 2004 to December 2008. GBS isolates from blood and cerebrospinal fluid (CSF) from infants with invasive disease, identified as part of routine medical care, were retrieved from the laboratory. The isolates were stored at −70°C in a broth containing skim milk, tryptone, glucose and glycerol (STGG) at the Respiratory and Meningeal Pathogens Research Unit., Johannesburg, South Africa, where serotyping was subsequently undertaken.

### Latex agglutination capsular serotyping

Capsular serotyping was performed with the latex agglutination method with specific antisera against types Ia, Ib and II to IX CPS antigens (Statens Serum Institute, SSI, Sweden) as previously described by Slotved *et al* [14]. Isolates that were reactive to sera against serotype III were further tested with sera against serotype VI to test for cross reactivity. Isolates that tested negative with all antisera were designated as serologically non-typeable and further typed by PCR. The GBS strains Ia (SSI-615), Ib (SS-618), II (SS-619), III (SS-620), IV (SS-1243), V (SS-1168), VI (SS-1354), VII (SS-1355), VIII (SS-1356), and IX (27412) were used as serotype specific control strains.

### Molecular capsular typing

Isolates designated as non-typeable by latex agglutination were characterized by a molecular capsular typing assay. DNA was extracted with the QIAamp DNA mini kit (Qiagen GmbH, USA) as per manufacture's recommendation. Capsular typing was performed with a singleplex PCR method for serotypes Ia, Ib, II, II, IV and V using primer sequences reported by Poyart *et al* [15]. The gene encoding *dlts* was used as a positive control for GBS identification.

### Statistical analysis

Data were analyzed using Graphpad Prism version 4.01 and STATA version 8.0. Serotype distribution between maternal and neonatal colonizing strains, and strains causing invasive disease was determined by a two tailed Fisher's exact test. Logistic regression was used to determine the association of frequency of serotypes in relation to the timing of onset of infant sepsis and disease syndrome.

As maternal colonization is a primarily risk factor for early onset (<7days of age) GBS disease, analyses of the invasive potential of GBS isolates was restricted to episodes within 7 days of birth and serotype associated with late onset (≥7 days of age) were excluded. Invasive potential of individual serotypes was estimated by calculating odds ratio (OR) using serotype III as a fixed reference serotype, since it has been consistently shown to be the most prevalent serotype in both colonizing and invasive disease isolates. This method has an advantage over calculating OR by reference to all other serotypes because the resulting estimate is a robust measure of invasive potential. The relative serotype-specific invasive potential was calculated based on the formula of odds ratio (OR)  =  (ad)/(bc) as described by Brueggemann *et al* [16]. Where “a” is the number of early onset isolates of a specific serotype; “b” is the number of early onset isolates of serotype III; “c” is the number of that specific serotype from maternal colonization isolates; and “d” is the number of serotype III from maternal colonization isolates. Associated 95% confidence intervals (95% CI) were estimated. Where two isolates of the same serotype were obtained from the same infant, only one isolate was included in the analysis.

## Results

Overall, GBS was identified from the vagina of 551 (21.5%) of 2 561 mothers and from the skin/mucosal surface of 402 (15.8%) of 2 542 newborns born mothers who were swabbed. GBS was identified in 289 (52.5%) newborns of the 551 mothers who were identified to be vaginally colonized by GBS during labor. In addition, GBS was also identified in a further 113 (5.6%) newborns born to 2,010 mothers in whom GBS was not detected on vaginal swabbing.

A total of 284 GBS isolates were obtained from 282 infants with invasive disease. These included 222 from blood (77.5%), and 62 from CSF (21.1%). GBS isolates were obtained from blood and CSF from two infants. The age distribution of infants with invasive isolates included 137 (48.2%) under 7 days of age, 108 (38.0%) between 7 to 90 days of age and 39 (13.7%) from children older than 90 days of age.

### Serotype distribution of maternal and newborn colonizing isolates

Serotyping was done on 541 (98.2%) of 551 available isolates obtained from colonized mothers and on 396 (98.5%) of 402 available isolates from colonized newborns. There were no differences in the serotype distribution (data not shown) between isolates from HIV-infected women (n = 119) compared to HIV-uninfected women (n = 418; P = 0.55). A total of 106 (19.6%) maternal and 76 (19.2%) newborn colonizing GBS isolates were serologically non-typeable (Table 1). Molecular capsular typing was successful in identifying a capsular serotype gene in 173 (95.1%) of the 182 serologically non-typable colonizing isolates (Table 1). The frequency of serotype distribution between serologically typeable isolates compared to those only typable by PCR differed as follows: Ia (32.4% vs. 14.8%, respectively; P<0.0001), Ib (2.4% vs. 23.1%, respectively; P<0.0001), II (13.3% vs. 4.4%, respectively; P = 0.0004), III (40.3% vs. 22.5%, respectively; P<0.0001); IV (1.3% vs. 15.4%, respectively; P<0.0001) and V (10.3% vs. 14.8%, respectively; P = 0.09).

**Table 1:** Serotype distribution among maternal and newborn colonizing Group B streptococcus isolates.

Serotype	Maternal colonization isolates			Neonatal colonization isolates			P valueComparing total of maternal vs. newborn serotypes
	Latexno = 541(%)	PCR^3^no = 106	Totalno = 541	Latexno = 395	PCRno = 76	Totalno = 395	
Ia	146 (27.0)^1^	17 (16.0)	163 (30.1)	98 (24.8)	10 (13.2)	108 (27.3)	P = 0.38
Ib	12 (2.2)	24 (22.6)	36 (6.7)	6 (1.5)	18 (23.7)	24 (6.1)	P = 0.79
II	55 (10.2)	6 (5.7)	61 (11.3)	45 (11.4)	2 (2.6)	47 (11.9)	P = 0.84
III	177 (32.7)	25 (23.6)	202 (37.3)	127 (32.2)	16 (21.1)	143 (36.2)	P = 0.73
IV	5 (0.9)	15 (14.2)	20 (3.7)	5 (1.3)	13 (17.1)	18 (4.6)	P = 0.51
V	40 (7.4)	15 (14.2)	55 (10.2)	38 (9.6)	12 (15.8)	50 (12.7)	P = 0.61
Non typeable	106 (19.6)	4 (3.8)		76 (19.2)	5 (6.6)		P = 0.22

1 Molecular serotype identification by PCR was done by primers targeting serotypes 1a to V.

Overall, of the colonizing isolates, serotype III was the most common in mothers (37.3%) and newborns (36.2%) (Table 1). Collectively, serotypes Ia, Ib and III accounted for 74.1% (401/541) of maternal and 69.6% (275/395) of newborn colonizing isolates. There were no differences in the relative frequencies of any of the serotypes between colonized mothers and colonized newborns. Isolates were available for serotyping from 280 (96.9%) of 289 mother-newborn pairs who were both colonized with GBS, among which there was 90.7% (254/280) concordance in serotype between the mother and newborn isolates. In addition, invasive GBS disease as a result of serotype III (n = 3) and Ia (n = 3) occurred in six newborns less than 7 days of age in whom colonizing isolates were available from the mother and the newborn, of which the invasive serotype was identical to the colonizing serotype in all of these newborns.

### Serotype distribution of invasive GBS isolates

The dominant serotypes causing invasive disease in infants less than 7 days and in those aged between 7 to 90 days were serotype III (57.7% vs. 84.3%, respectively; P<0.0001) and serotype Ia (22.6% vs. 13.9%, respectively; P = 0.01). Together, these serotypes accounted for 80.3% of invasive GBS disease occurring in neonates less than 7 days of age and 98.2% of invasive disease occurring in infants aged 7 to 90 days. Serotypes III and Ia together accounted for 53.9% of invasive GBS isolates occurring in children older than 90 days of age. Individually, serotypes Ib, II, IV, and V accounted for less than 6% of invasive isolates in infants less than 7 days of age and in infants aged 7 to 90 days. No invasive isolate was reactive to sera against serotypes VI, VII, VIII and IX. After adjusting for the type of specimen from where the isolate was recovered (i.e. blood only vs. CSF) and age group, serotype III remained the dominant cause of invasive disease in infants aged 7 to 90 days (Adjusted odds ratio: 3.60; 95%CI 1.91–6.78; P<0.0001).

Serologically non-typeable isolates were less common from invasive GBS isolates (20/284; 7.0%) compared to maternal or newborn colonizing isolates. Genotypic serotyping by PCR was successful in attributing a serotype to all serologically non-typeable invasive isolates (Table 2).

**Table 2:** Serotype distribution among invasive disease isolates from early onset (EOS; <7 days of age), late onset diseases (LOD; 7 to 90 days of age) and in children older than 90 days.

Serotype	Early Onsetno = 137	Late Onsetno = 108	>90 days ageno = 39	P valueEOS vs LOD
Ia	31 [1]^1^ (22.6) 2	15 [1] (13.9)	9 [0] (23.1)	P = 0.99
Ib	7 [2] (5.1)	0 [0] (0)	6 [2] (15.4)	P = 0.009
II	7 [2] (5.1)	0 [0] (0)	6 [2] (15.4)	P = 0.009
III	79 [3] (57.7)	91 [3] (84.3)	12 [1] (30.8)	P<0.0001
IV	5 [1] (3.6)	0 [0] (0)	0 [0] (0)	P = 0.07
V	8 [0] (5.8)	2 [1] (1.9)	6 [2] (15.4)	P = 0.19
NT	0	0	0	

1 Figure in squared parenthesis indicates number of isolates which were serologically non-reactive but typed by PCR.

2 Figure in rounded parenthesis is a percentage.

### Estimates of the relative invasive potential of serotypes

There were significant differences in the serotype distribution between invasive isolates from neonates younger than 7 days and maternal colonizing isolates. A higher proportion of newborn invasive isolates in those less than 7 days age were serotype III (79/137; 57.7%) compared to maternal colonizing isolates (202/537; 37.6%; P<0.0001). Conversely comparing invasive isolates in newborns under 7 days to maternal colonizing isolates, serotype II [7/137 (5.1%) *vs.* 61/537 (11.3%); respectively, P = 0.0008] and serotype V [8/137 (5.8%) *vs*. 55/537 (10.2%), respectively; P = 0.014] were less common as invasive isolates (Table 3).

**Table 3:** Estimation of invasive potential of Group B streptococcus (GBS) serotypes in different countries.

Serotype	Country					
	South Africa	Portugal [34]	Israel [36]	Sweden [31]	Netherlands [37]	Taiwan [37]
Ia	163^a^/31^b^(0.49; 0.31–0.77)^c^	42/13(1.52; 0.63–3.67)	12/10(1.14; 0.41–3.18)	15/20(1.37; 0.61–3.10)	24/6(0.52; 0.63–3.67)	13/5(0.22; 0.07–0.70)
Ib	36/7(0.50; 0.22–1.18)	14/1(0.35; 0.04–2.93)	9/2(0.30; 0.05–1.57)	15/2(0.14 0.03–0.64)	ND	5/2(0.22; 0.04–1.27)
II	61/7(0.30 0.13–0.67)	46/8(0.85; 0.32–2.27)	23/14(0.83; 0.34–2.03)	13/4(0.32; 0.09–1.07)	ND	ND
III	202/78(1.00)	59/12(1.00)	26/191.00	36/35(1.00)	20/33(1.00)	19/34(1.00)
IV	(20/5)(0.65; 0.23–1.79)	6/1(0.81; 0.09–7.44)	ND	3/2(0.69; 0.11–4.36)	ND	ND
V	55/8(0.38; 0.17–0.83)	59/7(0.58; 0.21–1.59)	18/9(0.68; 0.25–1.85)	25/7(0.29; 0.11–0.75)	14/4(0.17; 0.05–0.60)	15/2(0.07; 0.02–0.36)

In our data two isolates of the same serotype (i.e III) were obtained from the same infant, and only one isolate was included in the analysis.

a Value indicates colonizing isolates.

b Value indicates neonatal invasive isolates.

c Value in parenthesis indicates OR and 95% CI.

d ND: No data for either invasive, colonization or both isolates.

## Discussion

To our knowledge this study provides the most in-depth insight of the serotype epidemiology of colonizing isolates in mother-newborn dyads and invasive GBS isolates in an industrializing country setting, and particularly from Africa. Although there are extensive data on maternal colonizing serotype distribution from industrialized countries [17], [18], there are limited data comparing serotype distribution from colonized mothers and their newborns from industrializing countries [19], [20]. The findings from our study are similar to those reported elsewhere, [5], [19], [20] and confirm that maternal GBS vaginal-colonization is commonly (52.5%) associated with infant colonization. The vertical transmission of GBS from mother to the newborn was corroborated by the high concordance of matching serotypes in the colonized mother-newborn dyads. Our study, however, also identified GBS in 113 (28.1%) of 402 colonized newborns born to mothers in whom GBS was not identified by vaginal swabbing.

The latter, as well as the incomplete, albeit high, concordance in serotypes distribution associated with colonization in mother-newborn dyads in our study may be due to the limitations of the study in the sensitivity of identifying maternal colonization, limitations in detecting concurrent multiple colonizing serotypes, or possibly acquisition of GBS from non-maternal sources during or soon after birth [21]. The exclusion of rectal swabs to detect GBS colonization may have compromised the sensitivity in detecting GBS colonization [22]. Previous studies have reported that the sensitivity of detecting GBS colonization in pregnant women by undertaking both rectal and vaginal swabs is 18.5%–51% higher compared to taking vaginal swabs alone [23]–[27] and suggests that maternal rectal colonization may be important source for acquisition of GBS in newborns. Additionally, the yield of GBS on vaginal swabbing undertaken after the onset of labor may have been affected by draining liquor following the rupture of the placental membranes. Consequently, our study provides a minimal estimate as to the prevalence of GBS colonization in pregnant women during labor, as well as the proportion of colonized newborns who have acquired GBS from their mothers.

The serotype distribution identified in invasive isolates in our study are consistent with other smaller studies on invasive isolates elsewhere from Sub-Saharan Africa such as in Malawi [28], Zimbabwe [29], and an earlier study at the same centre as this study from 1997–1998. [30]) The serotype data of invasive isolates from this study are nevertheless important as it confirms the absence of temporal variation in serotype distribution of invasive isolates over more than a decade in the study-setting. [30] This has positive implications for the design of serotype-specific polysaccharide vaccines for prevention of newborn GBS sepsis.

Serotype III was responsible for 69.4% of invasive GBS disease among infants under the age of 90 days, which is in keeping with other studies from Sub Saharan Africa (56.0–58.3%) [28], [30]. Serotype Ia was the second most frequently identified invasive serotype in infants under 90 days of age in our study (18.8%), consistent with previous studies from South Africa (28.7%) and Malawi (21%) [28], [30]. These findings are also consistent with serotype distribution data of invasive isolates in young infants from industrialized countries (44% to 65% for serotype III and 33% to 15% for serotype Ia) [31]–[34]. Overall, 85.4% and 98.2% of invasive isolates in children less than 7 days and those aged 7 to 90 days of age, respectively belonged to serotypes Ia, Ib or III, which are currently being considered as serotypes to be included in a polysaccharide-protein conjugate vaccine against GBS [6], [10]. Similarly a trivalent serotype-specific GBS vaccine including these serotypes would provide potential cover against at least 80% of invasive serotypes in young infants in USA and European countries [31]–[34].

Our data indicated that serotype III (OR = 2.29) was the most invasive of all studied serotypes based on a method of analysis described by Brueggemann *et al*. [35]. These data are also consistent with findings from Portugal (OR = 1.42) [34], Sweden (OR = 2.61) [31], and Israel (OR = 1.84) [36] where the same method was used. Due to this consistency we benchmarked invasive potential of other serotypes with type III as a referent serotype [16] as summarized in Table 3. The data consistently reports that serotype Ib, II and V, are less invasive than serotype III. However, the invasive potential of serotype Ia is variable across different sites. In South Africa, Taiwan and The Netherlands [37], serotype Ia was less invasive than type III, although more invasive in Portugal [34], Israel [36] and Sweden [31]. The reasons for variation in invasive potential of serotype Ia reported at different sites are unclear, however data from Portugal suggests that the high invasive index of this serotype can be attributed to a dominant clone (ST-23 and ST-24), suggesting that the underlying genotype can influence the invasive potential.

Serotype V was identified in a low proportion of invasive isolates (5.6%) in our study, which is consistent with earlier studies from South Africa (5.6%) and Malawi (4.0%) [28], [30]. In the United States, serotype V is responsible for a high proportion of the invasive cases among infants (28%) [38]. Similarly, a higher prevalence of serotype V has also been reported in studies from England and Wales (13%) [39].Recent identification of these historically uncommon circulating strains in both colonization and invasive disease isolates raises questions as to whether introduction of a vaccine composed of the most common invasive serotypes could cause a shift in serotype distribution in colonization, and thus disease. This warrants the need for ongoing international surveillance of invasive GBS serotypes in order to optimize GBS vaccine formulations.

Our study demonstrated differences between serotype epidemiology of isolates associated with invasive disease in newborns compared to maternal colonizing isolates. These findings corroborate the finding of other studies from industrialized country settings [31], [34], [36], [37]. Our study, however, clarified that the difference in serotype distribution between invasive and colonizing isolates is related to an increased invasive potential of certain GBS serotypes (i.e. III and Ia) rather than due to increased risk of acquisition of these by the newborn from the mother.

Despite the success of intrapartum antibiotic prophylaxis in reducing GBS associated neonatal sepsis in industrialized countries, the high costs and resource intensiveness is prohibitive in industrializing countries [40]. Further limitations to this intervention strategy include concerns about the emergence of antimicrobial resistance in GBS, and a lack of efficacy in reducing GBS sepsis in infants older than 7 days of age [41]. As a result, neonatal morbidity due to GBS remains a public-health problem. In Sub-Saharan Africa GBS neonatal disease occurs in 3.06 per 1,000 live births in South Africa [30], and 1.81 per 1,000 live births in Malawi [28]. Consequently, the agenda of maternal immunization with GBS vaccines aimed at either reducing maternal recto-anal colonization, or preventing disease in the newborns through transplacental antibody transfer, needs to be explored in settings with a high burden of GBS disease.
